# High-Throughput Genetic Screening of 51 Pediatric Cataract Genes Identifies Causative Mutations in Inherited Pediatric Cataract in South Eastern Australia

**DOI:** 10.1534/g3.117.300109

**Published:** 2017-08-23

**Authors:** Shari Javadiyan, Jamie E. Craig, Emmanuelle Souzeau, Shiwani Sharma, Karen M. Lower, David A. Mackey, Sandra E. Staffieri, James E. Elder, Deepa Taranath, Tania Straga, Joanna Black, John Pater, Theresa Casey, Alex W. Hewitt, Kathryn P. Burdon

**Affiliations:** * Department of Ophthalmology, School of Medicine, Flinders University, Adelaide, South Australia 5042, Australia; † Department of Haematology and Genetic Pathology, School of Medicine, Flinders University, Adelaide, South Australia 5042, Australia; ‡ Centre for Ophthalmology and Visual Science, University of Western Australia, Lions Eye Institute, Perth, Western Australia 6009, Australia; § Centre for Eye Research Australia, Royal Victorian Eye and Ear Hospital, Melbourne, Victoria 3002, Australia; ** Department of Surgery, University of Melbourne, Victoria 3010, Australia; †† Department of Ophthalmology, Royal Children’s Hospital, Melbourne, Victoria 3052, Australia; ‡‡ Ophthalmology Department, Women’s and Children’s Hospital, Adelaide, South Australia 5006, Australia; §§ Department of Paediatrics, University of Melbourne, Victoria 3010, Australia; *** Menzies Institute for Medical Research, University of Tasmania, Hobart, Tasmania 7000, Australia

**Keywords:** Ion Torrent, PGM, congenital cataract, pediatric cataract, massively parallel sequencing, Mutant Screen Report

## Abstract

Pediatric cataract is a leading cause of childhood blindness. This study aimed to determine the genetic cause of pediatric cataract in Australian families by screening known disease-associated genes using massively parallel sequencing technology. We sequenced 51 previously reported pediatric cataract genes in 33 affected individuals with a family history (cases with previously known or published mutations were excluded) using the Ion Torrent Personal Genome Machine. Variants were prioritized for validation if they were predicted to alter the protein sequence and were absent or rare with minor allele frequency <1% in public databases. Confirmed mutations were assessed for segregation with the phenotype in all available family members. All identified novel or previously reported cataract-causing mutations were screened in 326 unrelated Australian controls. We detected 11 novel mutations in *GJA3*, *GJA8*, *CRYAA*, *CRYBB2*, *CRYGS*, *CRYGA*, *GCNT2*, *CRYGA*, and *MIP*; and three previously reported cataract-causing mutations in *GJA8*, *CRYAA*, and *CRYBB2*. The most commonly mutated genes were those coding for gap junctions and crystallin proteins. Including previous reports of pediatric cataract-associated mutations in our Australian cohort, known genes account for >60% of familial pediatric cataract in Australia, indicating that still more causative genes remain to be identified.

Pediatric cataract is one of the leading causes of blindness in children. Approximately 200,000 children worldwide are blind from this condition (Chan *et al.* 2012). In industrialized countries, the incidence is 1–6 per 10,000 live births (Santana and Waiswo 2011). In Australia, the incidence is 2.2 per 10,000 live births, making the condition one of the most common causes of visual impairment in children (Wirth *et al.* 2002). Intrauterine infection, drug exposure, metabolic disorders, malnutrition, and heredity are known risk factors for pediatric cataract (Churchill and Graw 2011). Pediatric cataract is often referred to as congenital or infantile cataract when it presents at birth or in the first year of life, and juvenile cataract when it presents later during childhood (Yi *et al.* 2011).

Around 25–33% of pediatric cataracts are inherited (Santana *et al.* 2009). It is thought that 28% of bilateral pediatric cataract cases have a genetic basis while only 2% of unilateral cases are genetic (Rahi and Dezateux 2001; Santana and Waiswo 2011). Genetic and clinical heterogeneity have been reported in inherited pediatric cataracts (Lorenz 2007). Inherited cataracts can be transmitted as autosomal recessive, autosomal dominant, or X-linked traits, with autosomal dominant being the most common mode of inheritance. They can be isolated (nonsyndromic) or combined with other phenotypic features (syndromic) (Hejtmancik 2008).

Changes in lens development including lens fiber cell differentiation, protein solubility and stability, and defects in lens structure can lead to the development of cataract (Huang and He 2010). Mutations in genes involved in these functions have been reported to cause pediatric cataract. The known genes include at least 10 crystallin genes (Churchill and Graw 2011; Berry *et al.* 2001; Kannabiran *et al.* 1998; Riazuddin *et al.* 2005; Willoughby *et al.* 2005; Litt *et al.* 1997, 1998; AlFadhli *et al.* 2012; Heon *et al.* 1999), as well as membrane proteins [*MIP*, *LIM2* (Pras *et al.* 2002; Berry *et al.* 2000)]; gap junction proteins [*GJA8*, *GJA3* (Beyer and Berthoud 2014)], cytoskeletal proteins [*BFSP1*, *BFSP2* (Ramachandran *et al.* 2007; Jakobs *et al.* 2000)]; stress response genes [*HSF4* (Bu *et al.* 2002)], cell signaling proteins [*EPHA2* (Shiels *et al.* 2008)], and transcription factors [*PITX3*, *PAX6*, *EYA1*, *FOXE3*, *VSX2*, *FTL* and *MAF* (Churchill and Graw 2011; Burdon *et al.* 2007; Shiels *et al.* 2007; Santana and Waiswo 2011)]. Crystallin and gap junction protein-encoding genes are the most commonly reported classes of genes for nonsyndromic pediatric cataracts, accounting for 50 and 25% of reported mutations, respectively (Hejtmancik 2008).

The large number of genes known to cause pediatric cataract and the limited genotype-phenotype correlations make clinical testing using traditional sequencing technologies challenging and expensive. Massively parallel (next-generation) sequencing (MPS) technologies are now accessible and are cost-effective tools to screen many candidate genes in parallel. In this study, we screened our repository of South Eastern Australian individuals with familial pediatric cataract for mutations in known causative genes using the Ion Torrent Personal Genome Machine (PGM). We hypothesized that a significant proportion of familial pediatric cataract cases would be accounted for by mutations in known genes, and that screening genes in parallel would be an effective method for genetic testing in this heterogeneous disease.

## Materials and Methods

### Participants’ recruitment and DNA extraction

The study adhered to the tenets of the Declaration of Helsinki and was approved by the Southern Adelaide Clinical Human Research Ethics Committee, Adelaide, Australia, and the Royal Victorian Eye and Ear Hospital (RVEEH) Human Research and Ethics Committee, Melbourne, Australia. The probands in each family were recruited from the eye clinic at Flinders Medical Centre (Adelaide), the Women’s and Children’s Hospital (Adelaide), the Royal Children’s Hospital (Melbourne), or the Royal Victorian Eye and Ear Hospital (Melbourne). Written informed consent was obtained from all participants or their guardians if they were under 18 years old. A detailed family history was obtained and additional affected and unaffected family members were invited to participate in the study. An ophthalmologist examined all available family members.

Genomic DNA was extracted from either peripheral whole blood using a QiaAmp DNA Blood Maxi Kit (Qiagen, Hilden, Germany) or from saliva using an Oragene DNA saliva collection kit (DNA Genotek, Ontario, Canada) according to the manufacturers’ protocols. Participants were included in this study if they reported a family history of pediatric cataract and if a causative mutation had not previously been detected in the family by other means.

### Gene selection, primer design, library preparation, and sequencing

Fifty-one genes known to cause pediatric cataract in human or mouse were selected through review of the literature (Supplemental Material, Table S1 in File S1 (Chen *et al.* 2011; Churchill and Graw 2011; Hughes *et al.* 2011; Lachke *et al.* 2011; Aldahmesh *et al.* 2012; Pras *et al.* 2004; Azuma *et al.* 2000; Nonnenmacher *et al.* 2011; Jamieson *et al.* 2007; Shiels *et al.* 2007; Zhou *et al.* 2010; Ferda Percin *et al.* 2000; Reis *et al.* 2012). The strategy for generating the list included all genes covered in a review of pediatric cataract genes (Churchill and Graw 2011) except the mouse gene *gjf1*, which does not have a human homolog (*n* = 39). A search in PubMed using the terms “paediatric cataract” or “congenital cataracts” in combination with “genetic” or “gene” was used to include additional genes from more recent publications and those genes not covered by the review (*n* = 12). We focused on those genes known to be associated with nonsyndromic pediatric cataract, as this was the predominant phenotype in our cohort. In addition, we included some syndromic pediatric cataract genes known to cause a predominantly ocular syndrome (*e.g.*, *PAX6* and *PITX3*) or where pediatric cataract is a major diagnostic feature of the syndrome (*NHS*) as such genes may also contribute to nonsyndromic cataract (Gillespie *et al.* 2014).

PCR primers to amplify coding, 3′-, and 5′-untranslated regions of the 51 genes were designed with the Ion AmpliSeq Designer tool v1.22 (Life Technologies, www.ampliseq.com). The final design consisted of a total of 1216 amplicons ranging from 125 to 225 bp, covering 94.26% of the target sequence. Primers were supplied in two 100 nM pools (Life Technologies, Carlsbad, CA). Briefly, the concentration of genomic DNA was determined using the dsDNA HS Assay Kit on a Qubit fluorometer (Life Technologies), and libraries were prepared with the Ion AmpliSeq library kit version 2.0 according to manufacturer’s protocols. Libraries were prepared in two pools per individual, and the amplified pools were combined before partially digesting the primers and barcode adaptor ligation. The amplified library was diluted to 10 pM, and 25 μl of the diluted library was used for template preparation using Ion PGM Template OT2 200 Kit (Life Technologies) and the manufacturer’s protocol. The clonally amplified library was then enriched on an Ion OneTouch enrichment system. Samples were barcoded during library preparation using Ion Xpress Barcode Adapters 1–16 kit (Life Technologies) and pooled in groups of 3–5 during template preparation on the Ion OneTouch.

Libraries were quantified either with a Bioanalyzer 2100 (Agilent Technologies, Santa Clara, CA) using the High Sensitivity DNA Kit (Agilent Technologies), or by qPCR using the Ion library TaqMan quantification kit (Life Technologies). Sequencing was performed on an Ion Torrent PGM using The Ion PGM Sequencing 200 Kit v2 and an Ion 318 chip (Life Technologies).

Torrent Suite (version 3.6) was used to align reads to human genome reference sequence 19 (hg19). The Coverage Analysis plugin (v4.0-r77897) was used to calculate the number of mapped reads, the percentage of on-target reads, and the mean depth of reads. Variants were called using the Variant caller plugin (V4.0-r76860) with the germline algorithm [allele frequency of 0.15, minimum read quality of 10, and minimum coverage of 20 were set as cut-offs for both indels and single nucleotide polymorphisms (SNPs)]. For annotation, variant call format (VCF) files were uploaded to Ion Reporter V4.0 (https://ionreporter.lifetechnologies.com/ir/) using the Ion Reporter Uploader plugin for Torrent Suite (v4.1-r79929). Variants were prioritized for further analysis if they were predicted to be protein-changing, and were absent or rare with minor allele frequency (MAF) <1% in dbSNP137 (http://www.ncbi.nlm.nih.gov/projects/SNP/), the Exome Aggregation Consortium (ExAC) (http://exac.broadinstitute.org/), and gnomAD (http://gnomad.broadinstitute.org/). In addition, identified variants were compared with an in-house list of common sequencing errors previously detected with this gene panel.

### Validation, segregation analysis, and evaluating potential functional effects of mutations

Direct Sanger sequencing was used to confirm the detected protein-changing mutations in probands and to evaluate the segregation of the mutation in families. Forward and reverse primer sequences were designed using Primer3 (Untergasser *et al.* 2012; Koressaar and Remm 2007) and are listed in Table S2 in File S1.

PCR reactions of 20 μl final volume consisted of 1× Coraload PCR buffer (Qiagen), which gave a final concentration of 1.5 mM Mg^2+^, 0.1 mM dNTPs (Roche Diagnostics, Risch-Rotkreuz, Switzerland), 0.5 μM of each primer, 0.5 U Hot Star Plus Taq Polymerase (Qiagen), and 40 ng of gDNA. Five times Q Solution (Qiagen) was included at a final concentration of 1× as required, and water volume was adjusted accordingly. PCR was performed on a Palm Cycler (Corbett Life science, Qiagen) with one cycle at 95° for 5 min, followed by 30 or 35 cycles (Table S2 in File S1) at 95° for 30 sec, 57−65° (annealing temperature, Table S2 in File S1) for 30 sec, and 72° for 30 sec, and a final extension step at 72° for 5 min. To clean the PCR products for sequencing, 5 μl of PCR product, 2 μl shrimp alkaline phosphatase (SAP; 1 unit/μl) and 0.5 μl (20 units/μl) of exonuclease 1 (Exo1) (New England Biolabs, Massachusetts) were mixed. Reactions were incubated at 37° for 1 hr, followed by incubation at 80° for 20 min to inactivate the enzymes.

Sequencing reactions were prepared with the respective forward primer at 5 μM and purified PCR product at 10 ng/100 bp (*i.e.*, 30 ng for 300 bp product) combined with BigDye Terminator v3.1 (Life Technologies) and 5× Sequencing Buffer (Life Technologies), and made up to 20 μl with water. Reactions were taken through a cycle sequencing PCR protocol on a MasterCycler thermal cycler (Eppendorf, Hamburg, Germany). PCR extension products were purified using Agencourt CleanSeq Magnetic Beads and a SPRI plate, according to the manufacturer’s protocol (Beckman Coulter, California). Purified extension products were then resolved using POP-7 polymer on the 3130xl Genetic Analyzer (Life Technologies) in the Flinders Sequencing Facility (Flinders Medical Centre, Adelaide, Australia).

Sequence chromatograms of affected and unaffected individuals were compared to each other and the reference sequence using Sequencher v.5 (GeneCodes Corporation, Ann Arbor, MI).

Each confirmed segregating novel mutation was assessed for a potential functional effect on the predicted protein sequence using SIFT (Sorting Intolerant from Tolerant, http://sift.jcvi.org/) (Kumar *et al.* 2009) and Polyphen-2 (version 2.2.2; the default HumDiv model was used) (Adzhubei *et al.* 2010) (http://genetics.bwh.harvard.edu/pph2/). The conservation of each altered amino acid was calculated using PhyloP as implemented in Mutation Taster (http://www.mutationtaster.org/) and available through the University of California Santa Cruz (UCSC) genome browser. PhyloP values between −14 and +6 indicate conservation at individual nucleotides, ignoring the effects of their neighbors. Amino acid conservation across species was visualized using the Mutation Taster website. Clinical interpretation of genetic variants by the 2015 ACMG/AMP guideline was determined using InterVar (http://wintervar.wglab.org) (Li and Wang 2017; Richards *et al.* 2015).

### Screening novel or previously reported variants in control population

Novel variants were screened in 326 unrelated normal Australian controls recruited from Flinders Medical Centre, Adelaide, using the MassArray platform and iPlEX chemistry (Sequenom) at the Australian Genome Research Facility (Brisbane, Australia). Variants identified in families CRCH139, CSA133, and CSA95 were screened in controls using custom TaqMan SNP genotyping assays (Life Technologies) on a StepOne Plus real-time PCR instrument (Life Technologies) using standard manufacturer’s protocols. All variants reported in this study have been submitted to the ClinVar database (http://www.ncbi.nlm.nih.gov/clinvar/; ClinVar accessions SCV000297746–SCV000297762).

### Data availability

Table S1 in File S1 contains a list of reported pediatric cataract genes selected for sequencing. PCR primers used to validate novel or rare coding mutations detected by next-generation sequencing are listed in Table S2 in File S1. Table S3 in File S1 contains systemic features of the five participants with syndromic pediatric cataract included in the study. Figure S1 in File S1 shows amplicons with <20 fold coverage. Figure S2, A–C in File S1) shows protein sequence alignments demonstrating the conservation of the altered amino acid in families with causative mutations.

## Results and Discussion

We sequenced 51 known pediatric cataract genes in 33 unrelated probands using Ion Torrent MPS technology. Syndromic cataract was present in 5/33 (15%) probands (syndromic features described in Table S3 in File S1) while 28/33 (85%) probands had isolated pediatric cataract. Primers were designed for 1216 amplicons in the size range of 125–225 bp to cover the 51 known causative genes. In total, 154.1 kb of target sequence was included in the design process with amplicons designed for 94.3% of the target sequence (8.8 kb not covered). The presence of repetitive sequence, unacceptable GC content, and melting temperatures of the primers outside the optimal range were the main factors limiting primer design for target regions not covered.

**Figure 1:**
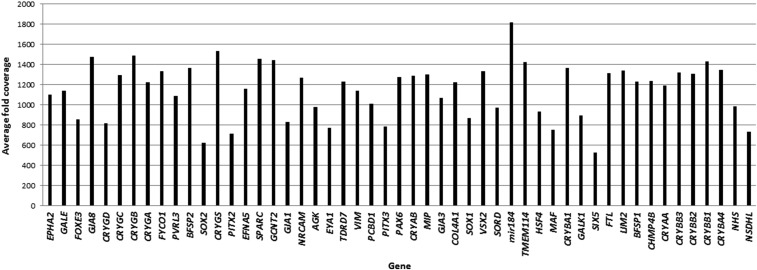
Average fold coverage of target genes sequenced from AmpliSeq libraries in 33 probands with pediatric cataract.

The mean number of mapped reads per sample was 1,536,538, with 91% of reads on target. A mean of 1155 reads was achieved per amplicon, with a coverage uniformity of 89%. Of all the amplicons, 96 and 91% were covered at least 20 and 100 fold, respectively. The average coverage per gene is shown in Figure 1. Of the 1216 amplicons, 30 amplicons (2%) across 17 genes were covered <20 fold (Figure S1 in File S1).

A total of 4726 variants were annotated (an average of 139 variants per individual). In total, 178 variants were absent/rare (MAF <1%) in publically referenced databases, of which 56 were nonsynonymous exonic variants. Twenty-three variants were selected for validation using Sanger sequencing after filtering out the variants in an in-house list of sequencing artifacts (33 variants). Seventeen variants were validated and six were false positives.

Of the 17 validated variants (Table 1), 14 appeared to be the likely cause of cataract in the respective families, accounting for 42% of the 33 screened probands. The validated mutations were considered to be pathogenic if the protein change was predicted to be pathogenic by SIFT (Kumar *et al.* 2009) and/or Polyphen-2 (Adzhubei *et al.* 2010), the variant segregated with the phenotype in the family, and was absent from all screened local controls. Two of the 17 validated variants did not segregate, and one was considered benign by both SIFT and Polyphen-2. All segregating mutations were highly conserved across species (Figure S2, A–C in File S1 and Table 1). We have used Polyphen-2 and SIFT predictions as a pathogenicity indicator guide; however, the predictions based on ACMG (Richards *et al.* 2015; Li and Wang 2017) guideline were also included for clinical purposes. The variants predicted to have uncertain significance under ACMG guideline look promising based on the evidence generated in this study; however, additional studies will be needed to confirm their pathogenicity.

**Table 1:** List of mutations detected in families

Family	Reported MAF in Public Databases	Novel/Known	Gene	Position in hg19	Nucleotide Change	Protein Change	PhyloP Score	Polyphen-2 (HumDiv)	SIFT	Segregation/Penetrance	Inheritance	ACMG
CSA95	0	Novel	*GJA3*	chr13:20717372	c.56C > T	p.(Thr19Met)	6.141	PD	D	Yes/ Full	AD	LP
CSA109	0	Novel	*GJA3*	chr13:20716962	c.466A > C	p.(Lys156Gln)	3.268	PD	D	Yes/ Full	AD	US
CRCH20	0	Novel	*GJA8*	chr1:147380155	c.73T > C	p.(Trp25Arg)	4.833	PD	D	Yes/ Incomplete	AD	LP
CSA125	0	Novel	*GJA8*	chr1:147380566	c.484G > A	p.(Glu162Lys)	5.784	PD	D	Yes/ Full	AD	US
CSA162	0	Known (Vanita *et al.* 2008) (Ma *et al.* 2016)	*GJA8*	chr1:147380216	c.134G > C	p.Trp45Ser	5.786	PD	D	Yes/ Full	AD	P
CSA159	0	Novel	*CRYAA*	chr21:44592307	c.440delA	p.(Gln147Argfs*48)	NA	NA	NA	Yes/ Full	AD	P
CRVEEH111	gnomAD: 0.00003231	Known (Khan *et al.* 2007) (Devi *et al.* 2008)	*CRYAA*	chr21:44589369	c.160C > T	p.(Arg54Cys)	4.982	PD	T	Yes/ Full	AD	P
CSA94	0	Novel	*CRYGS*	chr3:186257377-78	c.30_31delCTinsAA	p.(Phe10_Tyr11delinsLeuAsn)	NA	PD	D	Yes/ Full	AD	US
CRCH139	ExAc:0.00428 gnomAD: 0.003861 dbSNP:0.0022 (rs139353014)	Novel	*CRYGA*	chr2:209027941	c.239G > A	p.(Arg80His)	0.799	PD	T	Yes/ Incomplete	AD	LB
	ExAc: 0.001816 gnomAD: 0.001904 dbSNP: 0.0006 (rs79006549)	Novel	*PVRL3*	chr3:110841054	c.886A > C	p.(Asn296His)	4.027	PD	D	No		US
CSA133	0	Known (Litt *et al.* 1997)	*CRYBB2*	chr22:25627584	c.463C > T	p.(Gln155*)	NA	NA	D	Yes/ Full	AD	P
CRVEEH85	0	Novel	*CRYBB2*	chr22:25627684	c.563G > T	p.(Arg188Leu)	5.11	PD	D	Yes/ Full	AD	LP
	ExAC: 8.489 × 10^−6^ gnomAD:4.085 × 10^−6^	Novel	*BFSP2*	chr3:133191301	c.1136C > A	p.(Ala379Glu)	0.366	B	T	Yes/ Full		US
CRCH89	0	Novel	*GCNT2*	chr6:10626722	c.1091T > C	p.(Phe364Ser)	4.256	PD	D	Yes/ homozygous in cases/Full	AR	LP
CRCH136	0	Novel	*GCNT2*	chr6:10626796	c.1169_1172delATCA	p.(Asn388Arg*20)	NA	NA	NA	Yes/ heterozygous in cases/NA	AR	US
CSA131	0	Novel	*MIP*	chr12:56845225	c.631G > T	p.(Gly211*)	NA	NA	NA	Yes/Full	AD	US
	gnomAD: 0.00002439	Novel	*FYCO1*	chr3:46009288	c.1538G > A	p.(Arg513Gln)		B	T	No		US

MAF, minor allele frequency; PD, probably damaging; D, damaging; AD, autosomal dominant; LP, likely pathogenic; US, uncertain significance; P, pathogenic; NA, not applicable; T, tolerated; LB, likely benign; B, benign; AR, autosomal recessive. GenBank accession numbers are shown in Table S1 in File S1. Zero in second column indicates that the variant was not present in all three databases (ExAC, genome ID and dbSNP).

**Figure 2:**
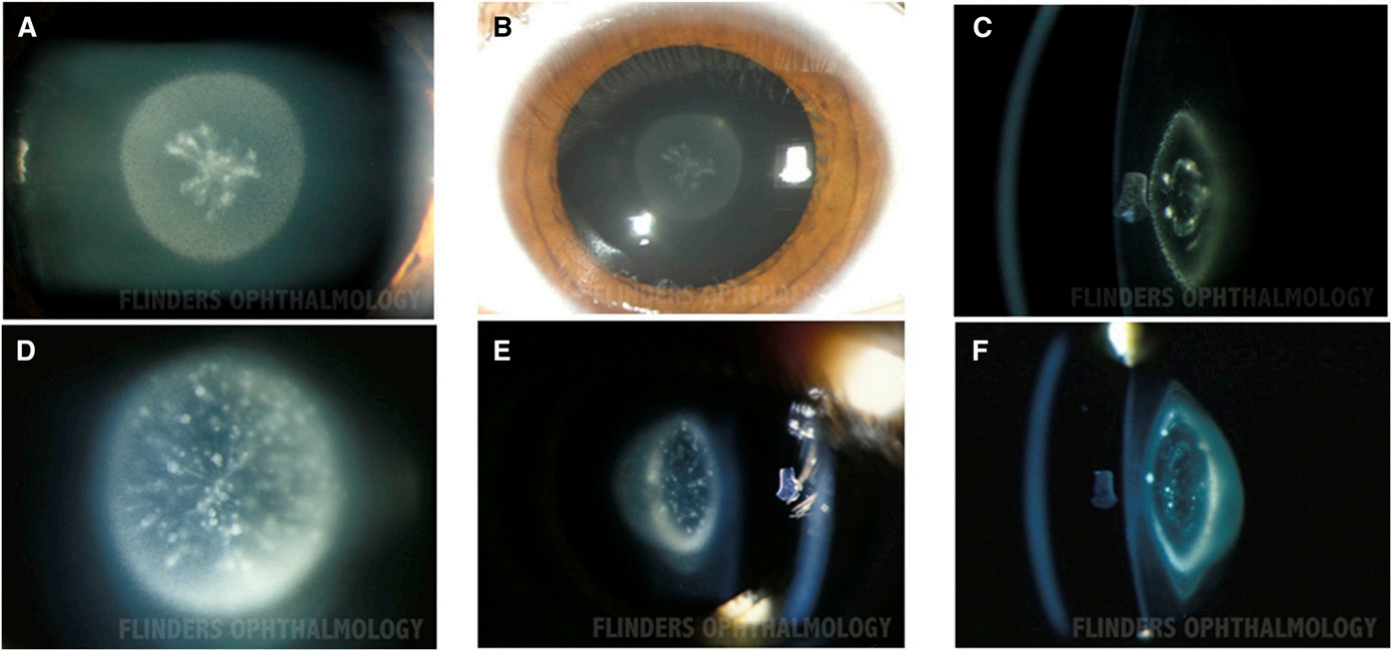
Phenotype of pediatric cataract in family CSA109 carrying causative mutations in *GJA3*. Photographs of individual CSA109.01 A–C show fetal nuclear cataract. D–F show fetal nuclear/lamellar cataract in individual CSA109.02.

In total, as shown in Table 1, we detected 11 novel mutations in eight different genes (*GJA3*, *GJA8*, *CRYAA*, *CRYBB2*, *CRYGS*, *CRYGA*, *GCNT2*, *CRYGA*, and *MIP*), three previously reported cataract-causing mutations in three different genes (*GJA8*, *CRYAA*, and *CRYBB2*). The phenotype in each of the 14 families is given in Table 2, and representative clinical photos where available are shown in Figure 2.

**Table 2:** Observed phenotypes in families with causative mutations identified in pediatric cataract associated genes

Family	Gene	Affected Members	Phenotype	Age at Diagnosis	Age at Surgery	Age at Surgery
					Right Eye	Left Eye
CSA95	*GJA3*	CSA95.01	—	0 yr	0 yr	0 yr
		CSA95.02	—	20 yr	—	—
CSA109	*GJA3*	CSA109.01	Fetal nuclear cataract	3 yr	—	—
		CSA109.02	Fetal nuclear/lamellar cataract	5 yr	16 yr	17 yr
CRCH20	*GJA8*	CRCH20.02	Bilateral congenital nuclear	—	35 yr	—
		CRCH20.07	Bilateral minor lens opacities	—	—	—
CSA125	*GJA8*	CSA125.01	Nuclear	10 yr	—	—
		CSA125.02	Posterior polar	—	6 yr	—
CSA162	*GJA8*	CSA162.01	—	—	—	—
		CSA162.02	—	—	—	—
CSA159	*CRYAA*	CSA159.01	Severe congenital	0 yr	1 mo	2 mo
		CSA159.02	Nuclear and cortical, blue-dot component: mild	19 yr	25 yr	25 yr
		CSA159.04	Lamellar: mild	4 yr	NA	NA
CRVEEH111	*CRYAA*	CRVEEH111.01	Bilateral	—	—	—
		CRVEEH111.04	Bilateral	—	17 mo	17 mo
		CRVEEH111.05	Central, anterior polar rider, faint nuclear opacity only	—	—	—
		CRVEEH111.06	Central nuclear opacity	—	—	—
CSA94	*CRYGS*	CSA94.01	Lamellar cortical-nuclear clear	6 yr	6 yr	5 yr
		CSA94.02	Cortical	4 yr	6 yr	5 yr
		CSA94.03	Lamellar	2 yr	3 yr	4 yr
		CSA94.04	Lamellar	2 yr	5 yr	5 yr
CRCH139	*CRYGA*	CRCH139.02	Congenital	—	—	—
CSA133	*CRYBB2*	CSA133.01	—	—	—	—
		CSA133.03	—	—	—	—
CRVEEH85	*CRYBB2*	CRVEEH85.01	Congenital	—	—	—
		CRVEEH85.02	Congenital	2–3 yr	3 yr	3 yr
		CRVEEH85.03	Congenital	—	—	—
CRCH89	*GCNT2*	CRCH89.01	Bilateral congenital	—	3 wk	3 wk
		CRCH89.02	Bilateral congenital	—	1 yr	1 yr
		CRCH89.05	Bilateral congenital	—	—	—
		CRCH89.07	Bilateral congenital	—	—	—
CRCH136*^a^*	*GCNT2*	CRCH136.01	Bilateral dense central opacity	—	—	—
		CRCH136.02	Bilateral dense central opacity	—	—	—
CSA131	*MIP*	CSA131.01	White dots	20 yr	NA	NA
		CSA131.02	White dots	22 yr	NA	NA
		CSA131.04	Cortical and nuclear sclerotic, multiple cortical dots as well as anterior cortical spokes	45 yr	46 yr	46 yr

Missing data are indicated by “—”. NA indicates the individual has not had surgery to date.

a One heterozygous deletion detected in affected members of this family with autosomal recessive inheritance pattern.

**Figure 3:**
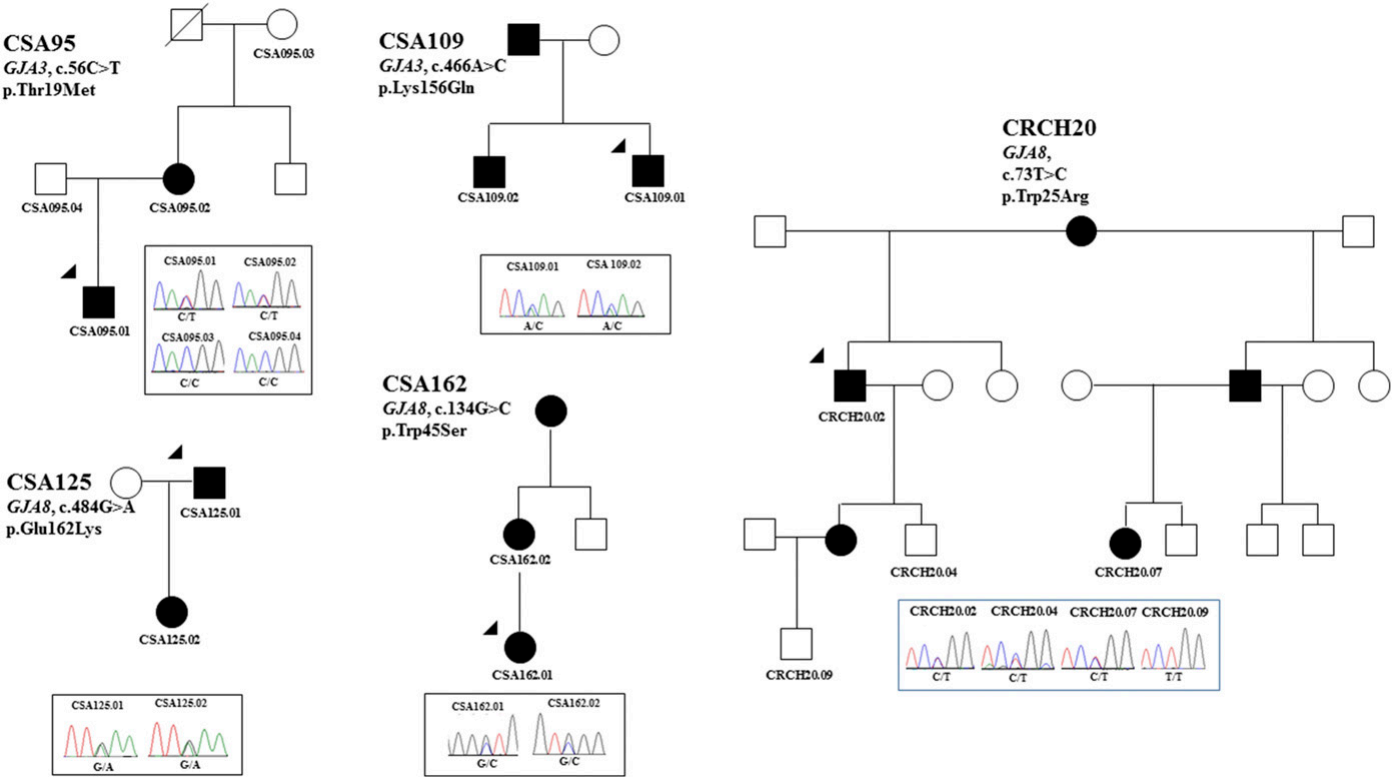
Pedigree and Sanger sequencing analysis of families with variants in gap junction genes (*GJA3* and *GJA8*). The chromatograms below each pedigree show the sequence detected via Sanger sequencing for each variant in families, and the gene names and mutation at cDNA and protein level have been mentioned on each pedigree. The penetrance of mutations in family CRCH20 (*GJA8*, c.73T > C) is incomplete. The arrowheads indicate the proband sequenced on the gene panel by AmpliSeq. Solid circles indicate affected females and solid squares show the affected males.

We identified novel mutations in two gap junction genes (*GJA3* and *GJA8*) in five families. Of the two families (CSA95 and CSA109) with mutations in *GJA3*, phenotypic information was not available for family CSA95, but variant p.Thr19Met was predicted to be pathogenic and segregated in the two affected individuals (Figure 3). Both tested individuals in family CSA109 had fetal nuclear lamellar cataracts (Figure 2) and the variant p.(Lys156Gln) was predicted to be damaging and segregated in two affected siblings. The affected father was not available for testing.

Two of the three families with *GJA8* mutations (CSA125 and CRCH20) had cataracts described as nuclear, with no phenotype information available for family CRCH162 (Table 2). In family CRCH20 the damaging mutation [p.(Trp25Arg)] segregated in two generations and appeared to have incomplete penetrance, as individual CRCH20.04 carries the mutation but as yet does not have cataract. Mutations in families CSA162 [p.(Trp45Ser)] and CSA125 [p.(Glu162Lys)] were inherited from the affected mother and the affected father, respectively (Figure 3), and segregated with the disease.

**Figure 4:**
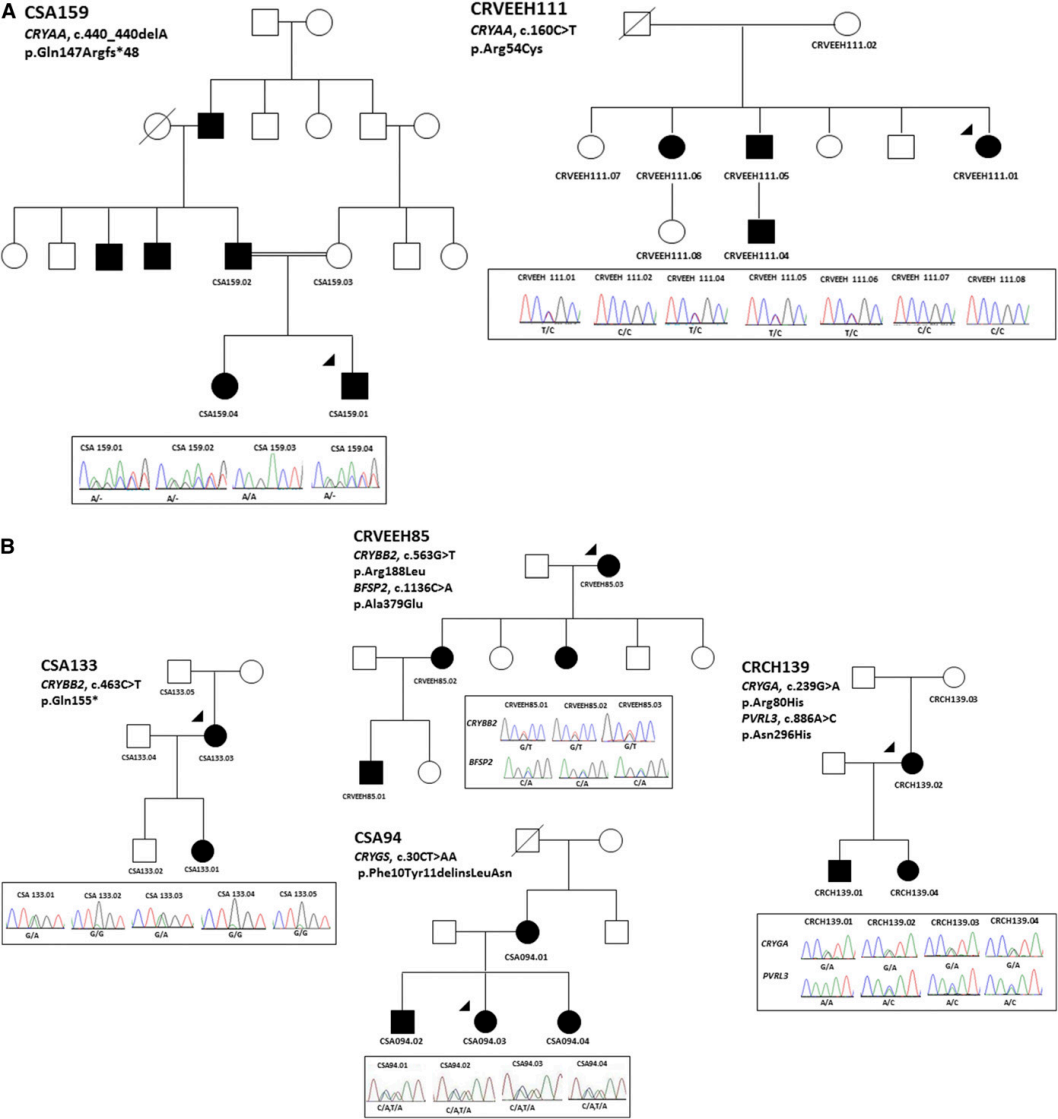
Pedigree and Sanger sequencing analysis of families with variants in (A) α crystallins (*CRYAA*); (B) β and γ crystallins (*CRYBB2*, *CRYGA*, and *CRYGS*). The penetrance of mutations in family CRCH139 (*CRYGA*, c.239G > A) is incomplete. The variants in *PVRL3* in CRCH139 do not segregate with the phenotype. The segregating variant in *BFAP2* in CRVEEH85 was predicted to be nonpathogenic by both SIFT and Polyphen-2. The arrowheads indicate the proband sequenced on the gene panel by AmpliSeq. Solid circles indicate affected females and solid squares show the affected males. Diagonal lines indicate the person is deceased. The chromatograms below each pedigree show the Sanger sequencing result of each detected variant in family members. The gene names and mutation at cDNA and protein level have been mentioned on each pedigree.

Six mutations were identified in crystallin genes. A previously reported mutation (Khan *et al.* 2007; Devi *et al.* 2008) in *CRYAA* [p.(Arg54Cys)] was detected in family CRVEEH111 that segregated with the disease (Figure 4A). SIFT predicted this variant to be tolerated, but Polyphen-2 predicted it to be pathogenic (Table 1). Family CRVEEH111 had central nuclear cataract with varying severity in affected family members. The second mutation detected in *CRYAA* is a novel frameshift deletion [p.(Gln147Argfs*48)] in a consanguineous family (CSA159) displaying autosomal dominant inheritance (Figure 4A). The father and both children carried the mutation; however, the severity of the phenotype varied between affected members. The proband (CSA159.01) was diagnosed at birth and underwent cataract surgery at 1 month of age. His sister (CSA159.04) was diagnosed with a milder lamellar cataract with a similar appearance to that in the father.

One novel and one previously reported mutation were detected in *CRYBB2*. Three affected individuals from family CRVEEH85 carried a novel mutation [p.(Arg188Leu)]. These individuals also carried a variant in *BFSP2* [p.(Ala379Glu)]. However, this variant was reported to be benign by both SIFT and Polyphen-2 and was less conserved. It was therefore considered not pathogenic. A previously reported truncating mutation, p.(Gln155*) (rs74315489), in the *CRYBB2* gene was identified in two affected individuals in family CSA133. No information was available regarding the phenotype in this family. This variant has not been reported in normal populations and was not detected in our local controls, thus is likely pathogenic.

Three mutations were detected in two different γ-crystallin genes. Family CSA94 (Figure 4B) had a novel dinucleotide substitution (c.30_31delCTinsAA) resulting in the substitution of two amino acids [p.(Phe10_Tyr11delinsLeuAsn]) in *CRYGS* which was predicted to be damaging (Table 1). Affected members of this family had a juvenile onset cortical lamellar cataract and all the members had surgery by 6 yr of age (Table 2). Family CRCH139 had a missense variant [p.(Arg80His), rs139353014] in *CRYGA* segregating with the phenotype in three individuals. This variant was predicted to be damaging by Polyphen-2 and the residue was conserved across species. However, it had a MAF of 0.2% in dbSNP, 0.4% in the ExAC database, and 0.1% in our Australian controls. The variant was also present in an unaffected individual CRCH139.03 in this family (Figure 4B). The reduced penetrance is consistent with this variant being present in the population at lower frequency. A second variant was detected in this family in *PVRL3*; however, it did not segregate with the phenotype. Although it is not clear whether the rare *CRYGA* variant is responsible for the disease in this family, it remains the best candidate mutation observed to date in family CRCH139 and may represent a deleterious variant of lower penetrance.

**Figure 5:**
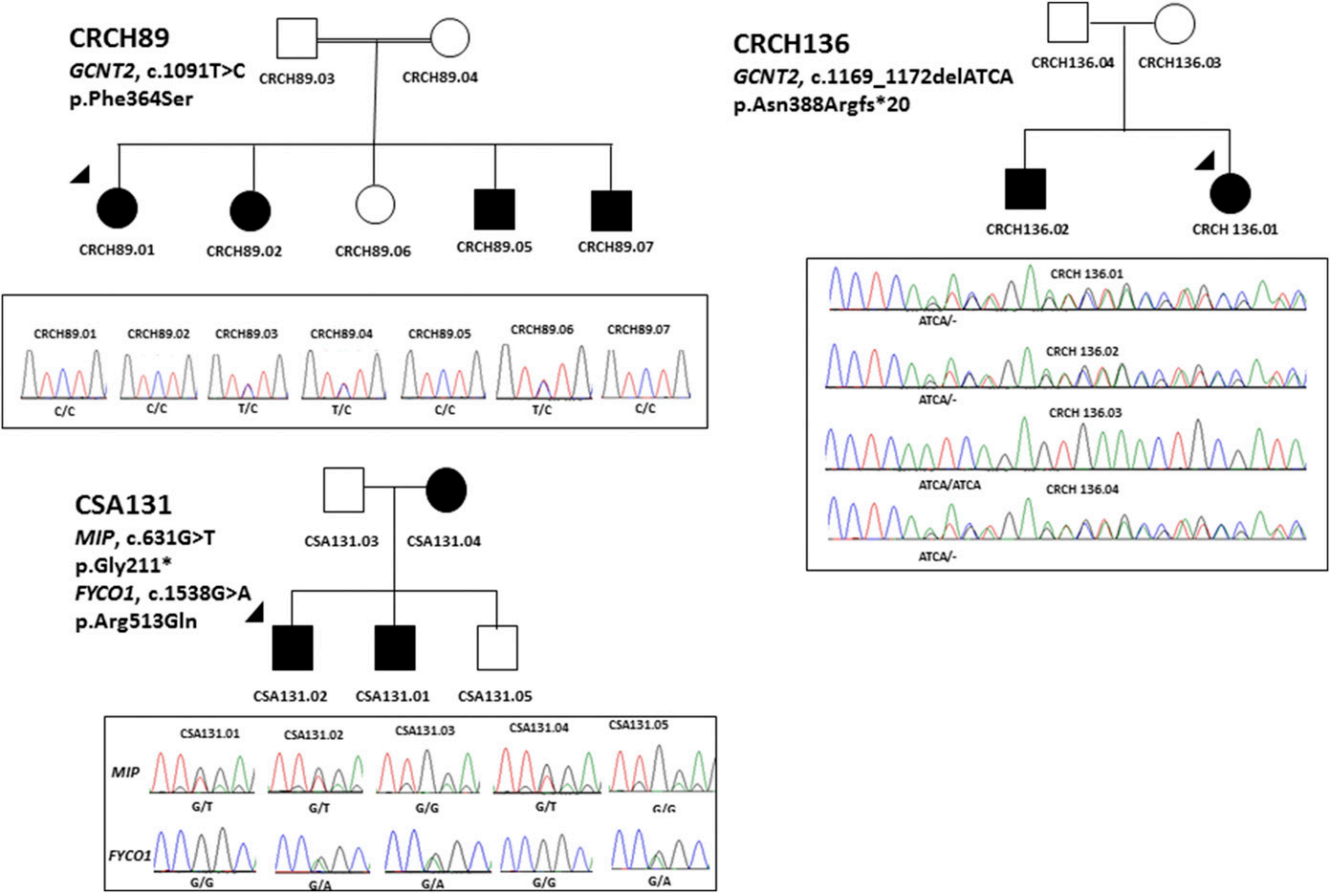
Pedigree and Sanger sequencing analysis of families with variants in *GCNT2* and *MIP*. The arrowheads indicate the proband sequenced on the gene panel by AmpliSeq. Solid circles indicate affected females and solid squares show the affected males. The double line in CRCH89 shows consanguinity. The chromatograms below each pedigree show the segregation analysis of the variants in families. The gene names and mutation at cDNA and protein level have been mentioned on each pedigree.

Families CRCH89 and CRCH136 displayed an autosomal recessive inheritance pattern of cataract. Affected members of the consanguineous family, CRCH89, were homozygous for a novel variant [p.(Phe364Ser)] in *GCNT2* predicted to be pathogenic (Figure 5). The four affected siblings all had bilateral pediatric cataracts with surgery in the first few weeks to 1 year of age (Table 2). A single heterozygous variant in *GCNT2* resulting in a premature stop codon [p.(Asn388Argfs*20)] was detected in family CRCH136 and was inherited from the unaffected mother. No other variant was identified in *GCNT2* in this family.

A segregating stop mutation [p.(Gly211*)] in *MIP* was detected in family CSA131 (Figure 5). The mother (CSA131.04) had cortical and nuclear sclerotic cataracts with multiple cortical dots, while two other affected members (CSA131.01 and CSA131.02) had anterior cortical spokes and white dot cataracts (Table 2). This family also carried a mutation in *FYCO1*; however, the latter variant was predicted to be benign and did not segregate with the disease.

Mutations meeting the criteria for potential pathogenicity were not identified in the remaining 19 families (57%) or the other 43 genes screened on this gene panel.

In this study, we used targeted massively parallel sequencing to identify genetic variants associated with inherited pediatric cataract. We identified likely causative variants in 42% of previously unsolved familial cases. We detected 11 novel mutations contributing to pediatric cataract, confirmed three previously reported mutations, and identified rare coding variants that may be important in the disease. We have previously identified mutations in genes included in this panel in other families in our repository (Sharma *et al.* 2008; Burdon *et al.* 2004a,b, 2007; Reches *et al.* 2007; Dave *et al.* 2013; Craig *et al.* 2003; McLeod *et al.* 2002; Javadiyan *et al.* 2016). When considered together with our earlier published work, these 51 genes account for 62% of familial pediatric cataract, a proportion comparable to that reported in a similar study of patients from the UK (Gillespie *et al.* 2014) and another Australian cohort (Ma *et al.* 2016).

The phenotypes observed in these families, where detailed information is available, are similar to previous reports of mutations in these genes. For example, we describe predominantly nuclear phenotypes related to mutations in the gap junction genes *GJA3* and *GJA8* (Shiels *et al.* 2010). Similarly, nuclear or total cataracts are observed in *CRYAA* mutation carriers (Khan *et al.* 2007; Devi *et al.* 2008) while mutations in *CRYBB2* give rise to cortical and lamellar cataracts (Devi *et al.* 2008; Faletra *et al.* 2013).

Homozygous or compound heterozygous mutations in *GCNT2* have been reported in families with autosomal recessive cataract (Borck *et al.* 2012). Affected individuals in family CRCH136 carry a single copy of a 4 bp frameshift deletion [p.(Asn388Argfs*20)] in *GCNT2*. Although segregation in this family is consistent with autosomal recessive inheritance (Figure 5), a second mutation in *GCNT2* or in any other gene in the panel could not be identified in the proband. The affected individuals had bilateral dense central opacities, similar to those reported by Borck *et al.* (2012) in other families with a homozygous mutation in this gene, and are also similar to those seen in family CRCH89 in which a homozygous *GCNT2* mutation was identified. It is possible that the second mutation in family CRCH136 was not detectable by the methods employed in this study, which may include partial gene deletions or mutations affecting noncoding regions. The possibility that this mutation does not contribute to the disease in this family cannot be excluded but is considered less likely.

This work highlights the not-infrequent occurrence of mutations with incomplete penetrance in pediatric cataract. We observed reduced penetrance with mutations in *GJA8* (CRCH20) and *CRYGA* (CRCH139). This suggests the involvement of modifier genes altering the penetrance of these mutations and the severity of the cataract; however, such genes have not yet been identified (Maeda *et al.* 2001). There have been previous reports of reduced penetrance with mutations in *GJA3* in families with total cortical (Devi *et al.* 2005) and nuclear lamellar pulverulent (Burdon *et al.* 2004b) cataracts. Furthermore, there has been one report of reduced penetrance of a mutation in *CRYBB2* associated with congenital zonular cataract (Santhiya *et al.* 2010).

The possible involvement of rare variants present in public databases in pediatric cataract pathogenesis is suggested by this study. Family CRCH139 has a segregating rare variant in *CRYGA* (rs139353014) with incomplete penetrance. Other factors, including high level of conservation at this residue and Polyphen-2 prediction of a functional effect, provide support for contribution of this mutation to cataract in this family. The incomplete penetrance is consistent with the presence of this variant at very low levels in public databases and our local controls. If this variant does not always lead to disease, it would be expected to accumulate in such databases. As public databases increase in size, and are generated from unscreened individuals, we expect that, increasingly, disease-causing variants will be found at low frequency in these resources. This scenario of disease-causing variants being represented in public databases of genetic variation due to the large numbers of individuals sequenced is well exemplified by the p.Gln368* mutation in the *MYOC* gene, which leads to primary open-angle glaucoma with high penetrance and is present in the 1000 Genomes database with a frequency of 0.06% as rs74315329. Such rare variation should not be automatically discounted when evaluating variants as pathogenic in disease cohorts. SIFT and ACMG predictions of benign for the Arg80His variant of CRCH139 make it difficult to comment on the pathogenicity of this variant without any functional studies. Other possibilities, such as the causative variant being intronic, in a novel not-yet-reported gene (or not included in this panel), or a large structural variant should not be ignored.

In the current study, 2% of amplicons were covered <20 fold, potentially limiting the ability to detect heterozygous mutations in these amplicons. By chance, the majority of the 16 genes containing these low coverage amplicons are involved in syndromic forms of pediatric cataract. In this study, only five probands had syndromic pediatric cataracts and no mutations were identified in these individuals. It may be that mutations were missed due to low coverage, or that these individuals have mutations in genes not targeted by this panel. Furthermore, it is important to comment that this study is not able to detect large insertions or deletions or any structural variants such as copy number variation (CNV) within screened genes. Such variants have been reported in the literature to be associated with the disease (Siggs *et al.* 2017; Burdon *et al.* 2007; Van Esch *et al.* 2007).

One of the main advantages of the AmpliSeq method on the Ion Torrent PGM is that it only requires 10–40 ng of DNA as starting material. Many of the DNA samples used in this study were >10 years old, and the limited quantity of DNA available was somewhat degraded. The successful sequencing of these samples and identification of likely causative mutations suggests that quality of DNA is not necessarily a crucial factor in obtaining reliable sequencing results using this methodology.

Pediatric cataract is a clinically and genetically heterogeneous condition, which makes accurate molecular diagnosis difficult. Genetic linkage analysis and candidate gene screening are the conventional methods for detecting disease-causing genes for familial diseases, but this approach is limited by the size of the families. In small families, as in this current cohort, gene identification has been difficult due to lack of power for linkage and the excessive cost of screening large numbers of candidate genes using traditional Sanger sequencing. MPS platforms are able to target numerous genes in parallel and are cost-effective tools for gene identification in heterogeneous conditions such as pediatric cataract. In this study, we aimed to determine the genetic contribution of the known pediatric cataract genes to inherited pediatric cataract in an Australian cohort. In our experience, both ophthalmologists and their patients are highly motivated to know the genetic basis of their disease. Developing genetic diagnostic panels increases the chance of obtaining a molecular diagnosis, which allows patients and their families to be better educated about the mode of inheritance, and facilitates more accurate genetic counseling about the risk of recurrence for future pregnancies, enabling discussion regarding possible reproductive options. For example, the parents in family CSA159 enrolled in this research study during a pregnancy with a desire to understand the likelihood of the child being affected with the severe disease observed in their son.

Although some degree of genotype/phenotype correlation is beginning to emerge for some pediatric cataract genes, the clinical evaluation of a patient is often insufficient to establish which genes are most likely involved in order to initiate specific genetic testing. This is particularly the case in historic samples where surgery was performed prior to enrolment in this study. These genotype-phenotype correlations are worthy of further study, as genetic counseling in the future should take account of the likely severity of affected status, the likelihood of systemic manifestation, and the chance of incomplete penetrance. Gene panel testing, as has been shown in previous studies (Gillespie *et al.* 2014) and here in an Australian cohort, is therefore an efficient way to rapidly determine the genetic cause of heterogeneous diseases such as pediatric cataract. In our cohort of cases, the chosen panel of 51 genes would cover 62% of causative mutations.

## Supplementary Material

Supplemental material is available online at www.g3journal.org/lookup/suppl/doi:10.1534/g3.117.300109/-/DC1.
