# Supplementary material for: High-Throughput Genetic Screening of 51 Pediatric Cataract Genes Identifies Causative Mutations in Inherited Pediatric Cataract in South Eastern Australia
Source: G3 (Bethesda). 2017 Aug 23;7(10):3257–68. doi: 10.1534/g3.117.300109 (PMC5633377; doi:10.1534/g3.117.300109)
Supplement: Supplementary file 1 [file 3257FileS1.docx]

**Supplementary material**

Table S1: List of reported paediatric cataract genes selected for sequencing

| **Name** | **Genbank accession** | **Locus** | **Reference** |
| --- | --- | --- | --- |
| *GALE* | NM_000403.3 | 1p36.11 | (Churchill and Graw, 2011) |
| GALK1 | NM_016905.2 | 17q25.1 | (Churchill and Graw, 2011)  (Stambolian, et al., 1995) |
| *NSDHL* | NM_015922.2 | [Xq28](http://www.omim.org/geneMap/X/740?start=-3&limit=10&highlight=740) | (Churchill and Graw, 2011) |
| *PCBD1* | NM_000281.3 | [10q22.1](http://www.omim.org/geneMap/10/233?start=-3&limit=10&highlight=233) | (Churchill and Graw, 2011) |
| *SORD* | NM_003104.5 | [15q21.1](http://www.omim.org/geneMap/15/147?start=-3&limit=10&highlight=147) | (Churchill and Graw, 2011)  (Vaca, et al., 1982) |
| *CRYAA* | NM_013501.2 | [21q22.3](http://www.omim.org/geneMap/21/117?start=-3&limit=10&highlight=117) | (Churchill and Graw, 2011)  (Litt, et al., 1998) |
| *CRYAB* | NM_009964.2 | [11q23.1](http://www.omim.org/geneMap/11/755?start=-3&limit=10&highlight=755) | (Churchill and Graw, 2011)  (Berry, et al., 2001) |
| *CRYBA1* | NM_009965.2 | [17q11.2](http://www.omim.org/geneMap/17/282?start=-3&limit=10&highlight=282) | (Churchill and Graw, 2011)  (Kannabiran, et al., 1998) |
| *CRYBB1* | NM_023695.2 | [22q12.1](http://www.omim.org/geneMap/22/129?start=-3&limit=10&highlight=129) | (Churchill and Graw, 2011)  (Willoughby, et al., 2005) |
| *CRYBB2* | NM_007773.3 | [22q11.23](http://www.omim.org/geneMap/22/113?start=-3&limit=10&highlight=113) | (Churchill and Graw, 2011)  (Litt, et al., 1997) |
| *CRYBB3* | NM_021352.3 | [22q11.23](http://www.omim.org/geneMap/22/112?start=-3&limit=10&highlight=112) | (Churchill and Graw, 2011)  (Riazuddin, et al., 2005) |
| *CRYGA* | NM_014617.3 | [2q34](http://www.omim.org/geneMap/2/745?start=-3&limit=10&highlight=745) | (Churchill and Graw, 2011) |
| *CRYGB* | NM_005210.3 | [2q34](http://www.omim.org/geneMap/2/744?start=-3&limit=10&highlight=744) | (Churchill and Graw, 2011)  (AlFadhli, et al., 2012) |
| *CRYGC* | NM_007775.2 | [2q33.3](http://www.omim.org/geneMap/2/739?start=-3&limit=10&highlight=739) | (Churchill and Graw, 2011) |
| *CRYGD* | NM_007776.2 | [2q33.3](http://www.omim.org/geneMap/2/738?start=-3&limit=10&highlight=738) | (Churchill and Graw, 2011)  (Heon, et al., 1999) |
| *CRYGS* | NM_017541.2 | [3q27.3](http://www.omim.org/geneMap/3/740?start=-3&limit=10&highlight=740) | (Churchill and Graw, 2011)  (Sun, et al., 2005) |
| *BFSP1* | NM_001195.3 | [20p12.1](http://www.omim.org/geneMap/20/90?start=-3&limit=10&highlight=90) | (Churchill and Graw, 2011)  (Ramachandran, et al., 2007) |
| *BFSP2* | NM_003571.2 | [3q22.1](http://www.omim.org/geneMap/3/542?start=-3&limit=10&highlight=542) | (Churchill and Graw, 2011)  (Jakobs, et al., 2000) |
| *COL4A1* | NM_001845.4 | [13q34](http://www.omim.org/geneMap/13/259?start=-3&limit=10&highlight=259) | (Churchill and Graw, 2011) |
| *GJA1* | NM_000165.3 | [6q22.31](http://www.omim.org/geneMap/6/696?start=-3&limit=10&highlight=696) | (Churchill and Graw, 2011) |
| *GJA3* | NM_021954.3 | [13q12.11](http://www.omim.org/geneMap/13/13?start=-3&limit=10&highlight=13) | (Churchill and Graw, 2011)  (Mackay, et al., 1999) |
| *GJA8* | NM_005267.4 | [1q21.2](http://www.omim.org/geneMap/1/811?start=-3&limit=10&highlight=811) | (Churchill and Graw, 2011)  (Shiels, et al., 1998) |
| *LIM2* | NM_005267.4 | [19q13.41](http://www.omim.org/geneMap/19/806?start=-3&limit=10&highlight=806) | (Churchill and Graw, 2011)  (Pras, et al., 2002) |
| *MIP* | NM_012064.3 | [12q13.3](http://www.omim.org/geneMap/12/435?start=-3&limit=10&highlight=435) | (Churchill and Graw, 2011)  (Berry, et al., 2000) |
| *NHS* | NM_001081052.1 | [Xp22.13](http://www.omim.org/geneMap/X/94?start=-3&limit=10&highlight=94) | (Churchill and Graw, 2011)  (Burdon, et al., 2003) |
| *NRCAM* | NM_001193582.1 | [7q31.1](http://www.omim.org/geneMap/7/480?start=-3&limit=10&highlight=480) | (Churchill and Graw, 2011) |
| *SPARC* | NM_003118.3 | [5q33.1](http://www.omim.org/geneMap/5/555?start=-3&limit=10&highlight=555) | (Churchill and Graw, 2011) |
| *VIM* | NM_203472.1 | [10p13](http://www.omim.org/geneMap/10/63?start=-3&limit=10&highlight=63) | (Churchill and Graw, 2011)  (Muller, et al., 2009) |
| *FOXE3* | NM_012186.2 | [1p33](http://www.omim.org/geneMap/1/452?start=-3&limit=10&highlight=452) | (Churchill and Graw, 2011)  (Bremond-Gignac, et al., 2010) |
| *HSF4* | NM_012186.2 | [16q22.1](http://www.omim.org/geneMap/16/432?start=-3&limit=10&highlight=432) | (Churchill and Graw, 2011)  (Bu, et al., 2002) |
| *MAF* | NM_005360.4. | [16q23.2](http://www.omim.org/geneMap/16/528?start=-3&limit=10&highlight=528) | (Churchill and Graw, 2011)  (Jamieson, et al., 2002) |
| *PAX6* | NM_000280.4 | [11p13](http://www.omim.org/geneMap/11/243?start=-3&limit=10&highlight=243) | (Churchill and Graw, 2011)  (Glaser, et al., 1994) |
| *PITX3* | NM_005029.3 | [10q24.32](http://www.omim.org/geneMap/10/416?start=-3&limit=10&highlight=416) | (Churchill and Graw, 2011)  (Semina, et al., 1998) |
| *SIX5* | NM_175875.4 | [19q13.32](http://www.omim.org/geneMap/19/651?start=-3&limit=10&highlight=651) | (Churchill and Graw, 2011) |
| *SOX1* | NM_005986.2 | [13q34](http://www.omim.org/geneMap/13/264?start=-3&limit=10&highlight=264) | (Churchill and Graw, 2011) |
| *SOX2* | NM_003106.3 | [3q26.33](http://www.omim.org/geneMap/3/700?start=-3&limit=10&highlight=700) | (Churchill and Graw, 2011) |
| *EPHA2* | NM_004431.3 | [1p36.13](http://www.omim.org/geneMap/1/156?start=-3&limit=10&highlight=156) | (Churchill and Graw, 2011)  (Shiels, et al., 2008) |
| *EFNA5* | NM_001962.2 | 5q21.3 | (Churchill and Graw, 2011) |
| *AGK* | NM_023538.2 | [7q34](http://www.omim.org/geneMap/7/605?start=-3&limit=10&highlight=605) | (Aldahmesh, et al., 2012) |
| *GCNT2* | NM_145649 | [6p24.3-p24.2](http://www.omim.org/geneMap/6/45?start=-3&limit=10&highlight=45) | (Pras, et al., 2004)  (Yu, et al., 2001) |
| *PVRL3* | NM_001243288.1 | [3q13.13](http://www.omim.org/geneMap/3/421?start=-3&limit=10&highlight=421) | (Churchill and Graw, 2011) |
| *EYA1* | NM_010164.2 | [8q13.3](http://www.omim.org/geneMap/8/278?start=-3&limit=10&highlight=278) | (Azuma, et al., 2000) |
| *FTL* | NM_010240.2 | [19q13.33](http://www.omim.org/geneMap/19/725?start=-3&limit=10&highlight=725) | (Nonnenmacher, et al., 2011) |
| *CHMP4B* | NM_176812.4 | [20q11.22](http://www.omim.org/geneMap/20/171?start=-3&limit=10&highlight=171) | (Shiels, et al., 2007) |
| *FYCO1* | NM_024513.3 | [3p21.31](http://www.omim.org/geneMap/3/194?start=-3&limit=10&highlight=194) | (Chen, et al., 2011) |
| *TMEM114* | NM_001146336.1 | [16p13.2](http://www.omim.org/geneMap/16/142?start=-3&limit=10&highlight=142) | (Jamieson, et al., 2007) |
| *TDRD7* | NM_014290.2 | [9q22.33](http://www.omim.org/geneMap/9/278?start=-3&limit=10&highlight=278) | (Lachke, et al., 2011) |
| *CRYBA4* | NM_021351.1 | [22q12.1](http://www.omim.org/geneMap/22/130?start=-3&limit=10&highlight=130) | (Zhou, et al., 2010) |
| *VSX2* | NM_182894.2 | [14q24.3](http://www.omim.org/geneMap/14/297?start=-3&limit=10&highlight=297) | (Ferda Percin, et al., 2000) |
| *PITX2* | NM_011098.3 | [4q25](http://www.omim.org/geneMap/4/389?start=-3&limit=10&highlight=389) | (Reis, et al., 2012) |
| *mir 184* | NR_038997.1 | Chr 15 | (Hughes, et al., 2011) |

| **Family** | **Gene** | **Mutation Position** | **Forward primer sequence** | **Reverse primer**  **sequence** | **Annealing temperature** |
| --- | --- | --- | --- | --- | --- |
| CSA 94 | *CRYGS* | chr3:186257377  chr3:186257378 | TGCCTCTCAAAATTTAATGTGAA | TGCTTTGTCCAAGGACCTAC | 57 |
| CSA159 | *CRYAA* | chr21:44592307 | GGCAGCTTCTCTGGCATG | GAGCCAGCCGAGGCCAATG | 65 |
| CSA109 | *GJA3* | chr13:20716962 | CGAGAACGTCTGCTACGACA | ATGAAGCAGTCCACCGTGTT | 57 |
| CRVEEH85 | *CRYBB2* | chr22:25627684 | CCCCTCGTTCACCCTCCCATCA | CACTGTGTCCAAGGTCACACAGCTAAGC | 57 |
| CSA125 | *GJA8* | chr1:147380566 | GTGCTGCAGATCATCTTCGT | GCTGCTCTACAGGCCTCTTC | 57 (35×) |
| CRVEEH111 | *CRYAA* | chr21:44589369 | GCTGACTGAGCAGCCTTCTT | GACGGAGCAAGACCAGAGTC | 57 |
| CSA131 | *MIP* | chr12:56845225 | GAAAGCAACATACAAACTAGTGCAA | CCCCTCCACGTAAACTCAGA | 57 (35×) |
| CSA162 | *GJA8* | chr1:147380216 | CCGCGTTAGCAAAAACAGAT | CAGCCGGAACTTCTTAGTGC | 57 |
| CRCH136 | *GCNT2* | chr6:10626796 | GGCTGAGACTGCACAATCAT | TTACGTAGCCAGGTCCTGAAG | 57 |
| CRCH89 | *GCNT2* | chr6:10626722 | GGCTGAGACTGCACAATCAT | TTACGTAGCCAGGTCCTGAAG | 57 |
| CRCH20 | *GJA8* | chr1:147380155 | TCTGCACAAAGGAAGCACTG | CTTTTGCGCTTCTCCTCCAT | 57 |
| CSA95 | *GJA3* | chr13:20717372 | GAGAAGCTGCCCATCAGC | GCGTGGACACGAAGATGA | 57 |
| CSA133 | *CRYBB2* | chr22:25627584 | AGAAAGCAGAGGCTCAGTGC | CAAAGACCCACAGCAGACAA | 60 |
| CRCH139 | *CRYGA* | chr2:209027941 | GGATGTTCCTTCCAGCTGAC | TGAACACTCATCCTGTGTTGG | 57 (35×) |

Table S2: PCR primers used to validate novel or rare coding mutations detected by next generation sequencing. Optimal PCR conditions for each pair are also given.

Table S3. Systemic features of the 5 participants with syndromic paediatric cataract included in the study.

| **Proband** | **Age at diagnosis** | **Systemic features** |
| --- | --- | --- |
| CSA119.01 |  | Retinitis Pigmentosa, coarctation of aorta |
| CSA132.03 | 0 | Hypospadias, vitamin D deficiency, behavioural issues |
| CSA128.01 | 8 | Seizures, lower limb weakness episode |
| CSA161.01 | 5 | Cleft palate, short stature |
| CSA158.01 | 5 | Mild left hemiplegia, spastic diplegia, mild expressive language delay |

Figure S1: Amplicons (names are given by Ampliseq designer) with less than 20 fold coverage. The bars show the average amplicon coverage of 33 individuals screened.

A


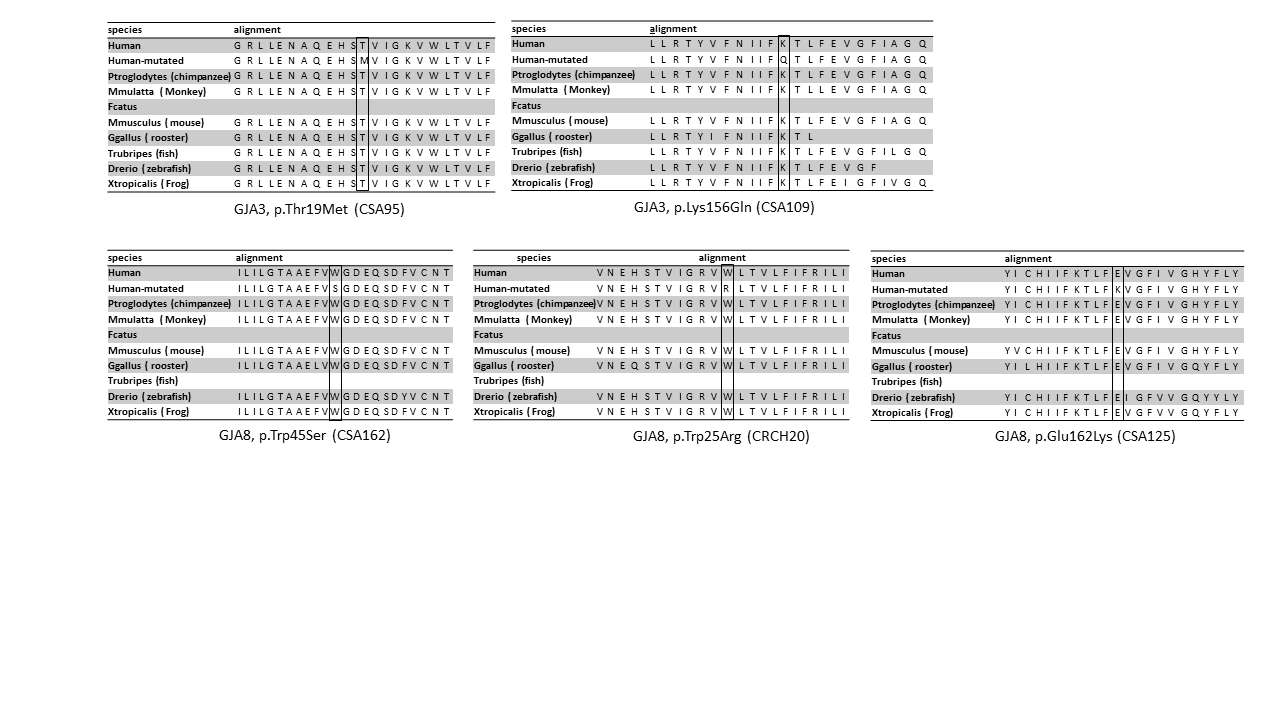


B


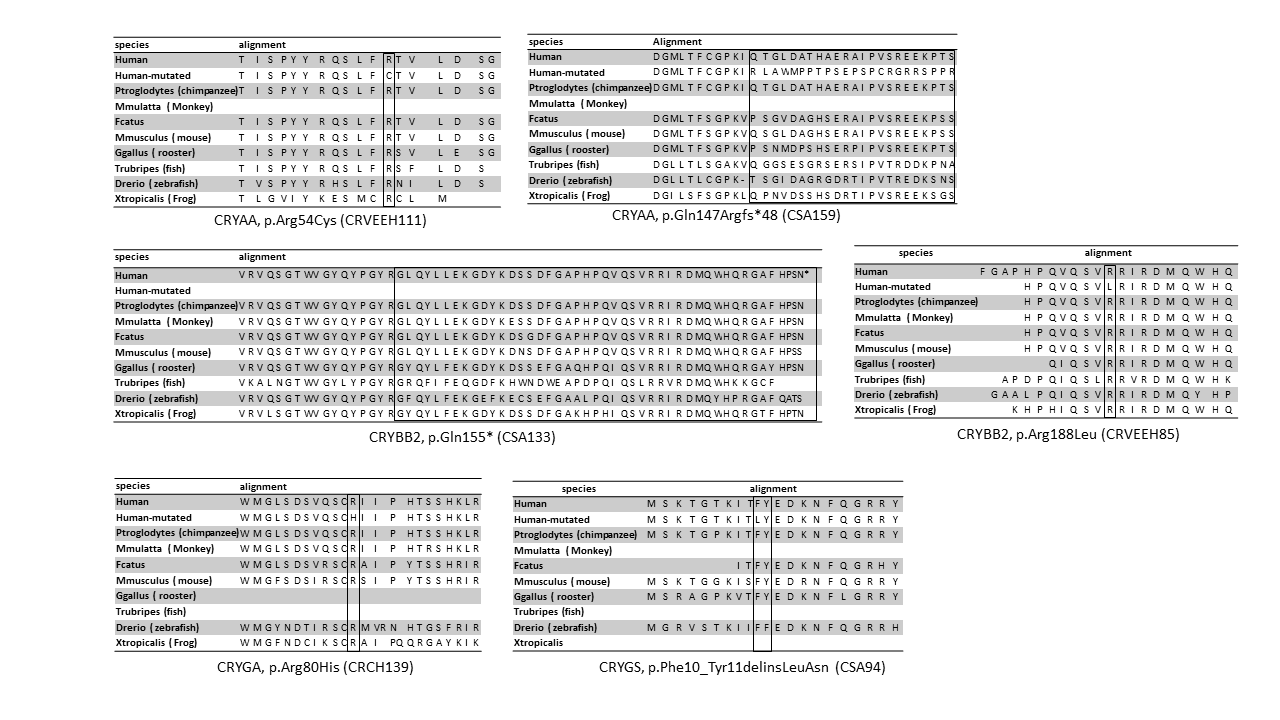


C


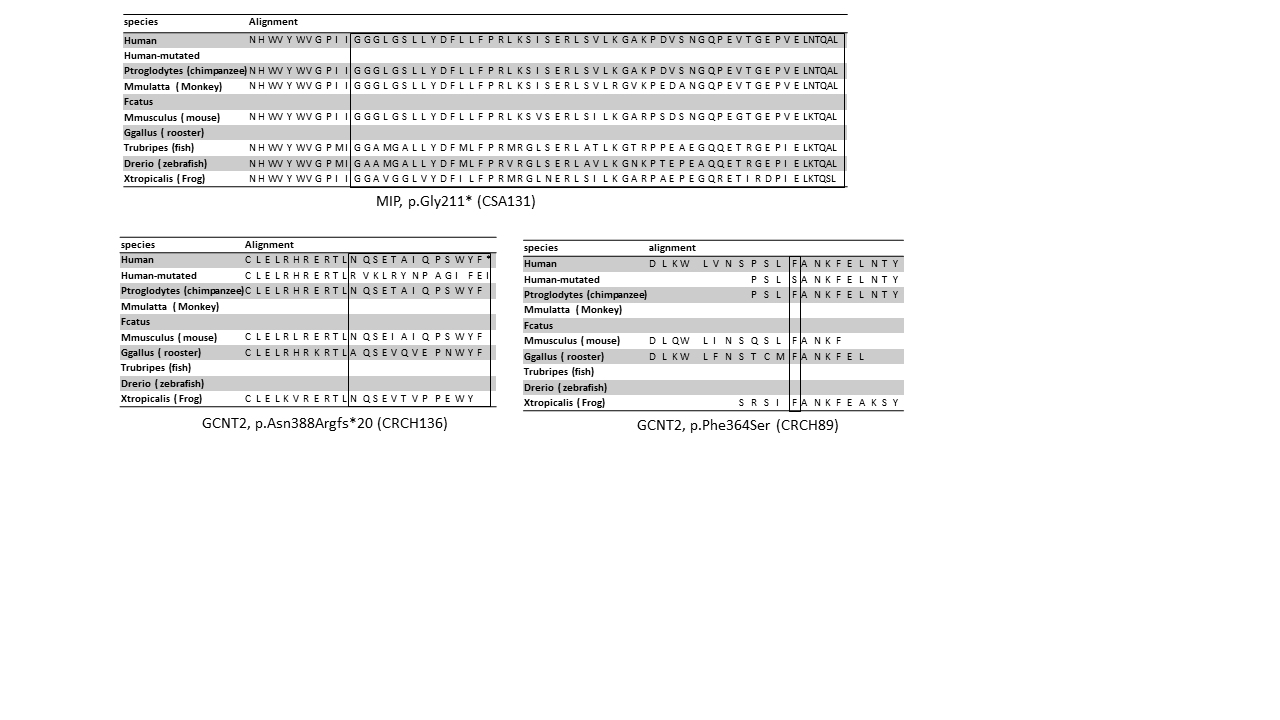


Figure S2: Protein sequence alignments demonstrating the conservation of the altered amino acid (boxed) in families with causative mutations. Families with protein alterations in A: Gap junction (GJA3 and GJA8); B: Crystallin (CRYAA, CRYBB2, CRYGA and CRYGS) and C: GCNT2 and MIP. The alignments are generated using Mutation Taster (<http://www.mutationtaster.org/>) and show alignments of 8 protein sequences to the human protein sequence. Both mutated and wild type human protein sequences are given beside sequences from other species. Empty rows mean that there is no homologue for a gene in that particular species. Family references for each mutation are shown in brackets under the alignments.
